# Synthesis of High-Molecular-Weight Polypropylene Elastomer by Propylene Polymerization Using α-Diimine Nickel Catalysts

**DOI:** 10.3390/polym16162376

**Published:** 2024-08-22

**Authors:** Lujie Gao, Hegang Ren, Yanhui Hou, Linlin Ye, Hao Meng, Binyuan Liu, Min Yang

**Affiliations:** 1 Hebei Key Laboratory of Functional Polymers, Institute of Polymer Science and Engineering, Hebei University of Technology, Tianjin 300401, China; 2 School of Material Science and Engineering, Guangdong University of Petrochemical Technology, Maoming 525000, China; 3 State Key Laboratory of Separation Membranes and Membrane Processes, School of Material Science and Engineering, Tiangong University, Tianjin 300160, China

**Keywords:** α-diimine nickel(II) catalyst, propylene polymerization, polypropylene elastomer, high molecular weight

## Abstract

The α-diimine late transition metal catalyst represents a new strategy for the synthesis of atactic polypropylene elastomer. Taking into account the properties of the material, enhancing the molecular weight of polypropylene at an elevated temperature through modifying the catalyst structure, and further increasing the activity of α-diimine catalyst for propylene polymerization, are urgent problems to be solved. In this work, two α-diimine nickel(II) catalysts with multiple hydroxymethyl phenyl substituents were synthesized and used for propylene homopolymerization. The maximum catalytic activity was 5.40 × 10^5^ gPP/molNi·h, and the activity was still maintained above 10^5^ gPP/molNi·h at 50 °C. The large steric hindrance of catalysts inhibited the chain-walking and chain-transfer reactions, resulting in polypropylene with high molecular weights (407~1101 kg/mol) and low 1,3-enchainment content (3.57~16.96%) in toluene. The low tensile strength (0.3~1.0 MPa), high elongation at break (218~403%) and strain recovery properties (S.R. ~50%, 10 tension cycles) of the resulting polypropylenes, as well as the visible light transmittance of approximately 90%, indicate the characteristics of the transparent elastomer.

## 1. Introduction

As a very popular plastic material, polypropylene (PP) is used for many applications, and can be classified into isotactic polypropylene (*i*PP), syndiotactic polypropylene (*s*PP), and atactic polypropylene (*a*PP). Among these, unlike the low-molecular-weight *a*PP obtained as a byproduct of *i*PP [1], amorphous high-molecular-weight *a*PP is a kind of elastomer material with growing industrial application prospects. Its beneficial elasticity and optical, antioxidant, and anti-aging properties make it widely used as an adhesive, compatibilizer, and additive in a series of polymer materials [2,3,4]. Only a few Ziegler catalysts [5], non-metallocene catalysts [6,7], and *C*_s_-, *C*_2v_- and *C*_1_-symmetric metallocene catalysts [8,9,10,11,12,13] are available for the synthesis of atactic polypropylene. Notably, metallocene catalysts are preferred for the preparation of high-molecular-weight atactic polypropylene elastomers, but they are challenging to synthesize and sensitive to moisture and oxygen [14].

In the 1990s, the α-diimine nickel/palladium complexes [14,15] attracted the attention of researchers due to their easy synthesis, efficient catalytic activity, and good tolerance to moisture and oxygen. Due to the fast “chain-walking” property of α-diimine catalysts, they can catalyze ethylene to produce branched polyethylene [16,17,18,19,20,21]. Formation of (1,ω) enchainment “chain straightening”, which has also been observed during the polymerization of α-olefin (e.g., 1-hexene, 1-octene, 1-decene) [22,23,24,25], results in ethylene units in the polymer chain. Some α-diimine catalysts are also used for olefin copolymerization to improve polyolefin properties [26,27,28,29,30,31]. When propylene is used as the monomer and catalyzed by the α-diimine catalyst, the resulting polymer exhibits a structure similar to that of the ethylene–propylene copolymer, as well as elastomeric properties [32,33,34,35,36,37,38,39,40,41,42,43]. Many studies have focused on the catalytic mechanism and the catalyst’s structure. The researchers found that during polymerization, in addition to the 1,2-insertion process, the 2,1-insertion and subsequent *β*-H elimination and reinsertion result in 1,3-enchainment of propylene [32,33,34,38]. At the same time, the chain-walking process caused by the α-diimine catalyst results in the microstructure of the polymer with long and short alkyl side-chains and an irregular main chain structure. These reactions can be controlled by adjusting the polymerization temperature. Although chain walking can be inhibited at low temperatures and 1,3-enchainment decreases, a low temperature will also lead to a decrease in molecular weight. For example, Coates’s group reported a series of *C*_2_-symmetric α-diimine Ni(II) complexes, which were shown to be highly regioregular and isoselective for propylene polymerization at low temperatures, whereas higher temperatures furnished regioirregular polypropylene composed of 1,2- and 1,3-enchainments [35,36]. With the decrease in temperature from 22 °C to −78 °C, the 1,3-enchainment content of polypropylene decreased from 56.2% to 0%, but the molecular weight was only 1/10 of that at room temperature (e.g., 57,100 vs. 5700 g/mol) [35].

On the other hand, it is an effective strategy to control the microstructures of polymers by modifying the catalyst structure while ensuring the high molecular weight of the polymer. The bulky *N*-aryl substituents and ligand backbone structures of α-diimine complex can inhibit the occurrence of the chain-transfer reaction and *β*-H elimination reaction, reduce the content of 1,3-enchainment, and increase the molecular weight of the polypropylene. The cyclophane backbone structure of the α-diimine nickel complex prepared by Guan’s group restricted C-H activation and improved the thermal stability of the catalyst, enabling the efficient preparation of polypropylene with 133 kg/mol of *M*_n_ at 50 °C [37]. Gao’s group prepared α-diimine nickel catalysts with a bulky camphyl backbone that exhibited some living characteristics and afforded the activity of 16 kg/molNi∙h and *M*_n_ of 54 kg/mol of polypropylene at 50 °C. Due to the camphyl backbone suppressing the potential rotation of the C–N bond by blocking the axial sites of metal centers, the catalysts even showed a medium activity of 14 kg/mol at 70 °C [39]. Chen’s group synthesized a series of dibenzhydryl-based α-diimine palladium [40] and asymmetric-naphthyl-substituted α-diimine nickel complexes [41]. They found that bulky steric hindrance substituents would slow down the chain-walking process, but excessively large substituents could also inhibit the coordination and insertion of propylene. At 30 °C, the highest molecular weight of polypropylene was 220 kg/mol and the content of 1,3-enchainment was low (27%). It also showed that it is possible to control 2,1-insertion by ligand design. Tang et al. synthesized a series of α-diimine nickel complexes with π–π stacking interaction [42]. The resultant polypropylenes had low 1,3-enchainment (23.27–34.78%). At 20 °C, the highest *M*_n_ of polypropylene was 350 kg/mol. However, when the temperature was above 40 °C, the performance of the catalyst decreased significantly. Dai and colleagues synthesized α-diimine palladium catalysts with flexible cycloalkyl substituents [43]. Bulky substituents inhibited the chain-walking process, resulting in moderate activity (10^4^ g/mol∙h) and polypropylene with a high *M*_n_, up to 125.2 kg/mol at 30 °C.

**Scheme 1:**
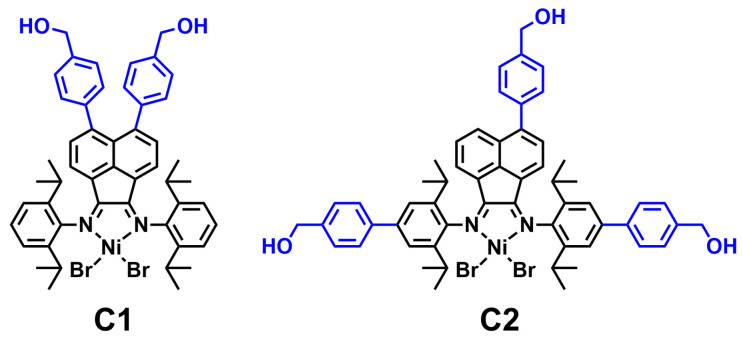
α-Diimine nickel(II) catalysts in this work.

Taking into account the properties of the material, enhancing the molecular weight of polypropylene at an elevated temperature by modifying the catalyst structure, as well as further increasing the activity of the α-diimine catalyst for propylene polymerization, are urgent problems to be solved. Our group designed and synthesized a series of α-diimine catalysts for ethylene polymerization, achieving good results in catalytic activity, thermal stability, and chain structure control of polyethylene [44,45,46]. Based on these results, two α-diimine nickel catalysts with multiple hydroxymethyl phenyl substituents were synthesized and used in propylene homopolymerization to prepare polypropylene elastomers (Scheme 1). The influences of catalyst structure and polymerization conditions on catalytic activity, as well as the chain structure, molecular weight, and stress–strain properties of polypropylene products, were investigated.

## 2. Materials and Methods

### 2.1. General Methods and Materials

All experiments sensitive to moisture and air were in conformity with the specification for a dry and pure argon atmosphere using the standard Schlenk technique. Toluene and hexane were used after reflux distillation in an Ar atmosphere using sodium. Dichloromethane (CH_2_Cl_2_) was obtained by reflux distillation after dehydrating with CaH_2_. Diethylaluminum chloride (AlEt_2_Cl, 1.0 mol/L in n-hexane) was purchased from Yanfeng Technology Co., Ltd. (Shenyang, China). Methylaluminoxane (MAO, 1.0 mol/L in n-hexane) and triethylaluminum (AlEt_3_, 1.0 mol/L in n-hexane) were purchased from Zesheng Technology Co., Ltd. (Anqing, China). High-purity (99.9%) propylene gas was purchased from Dongrun Specialty Gases Co., Ltd. (Tianjin, China). The remaining chemicals were obtained through commercial means with no purification.

### 2.2. Characterizations

The FT–IR spectra of the ligands and corresponding nickel complexes were obtained by pressing KBr pellets using a Thermo Nicolet 6700 spectrometer (Thermo Fisher Scientific, Waltham, MA, USA). The ^1^H NMR spectra of the ligands in CDCl_3_ at ambient temperature were acquired by Bruker DMX 400 MHz (Bruker Co., New Castle, DE, USA), with TMS as an internal standard; δ values are reported in ppm and J values in Hz. Elemental analysis was obtained using a Flash EA 1112 microanalyzer. The molecular weight *M*_w_ (weight average molar weight), *M*_n_ (number average molar weight), and distribution PDI (polymer dispersity index) of the polypropylene were determined in THF at 40 °C using the 1260 Infinity II system (Agilent Technologies, Inc., Santa Clara, CA, USA). The ^1^H and ^13^C NMR spectra of the structures of branched polypropylenes were obtained at 120 °C using deuterated 1,2-dichlorobenzene and TMS as the internal standard on the Bruker DMX 400 MHz system (Bruker Co., New Castle, DE, USA); δ values are reported in ppm. Differential scanning calorimetry (DSC) measurements were performed on a TA Instruments DSC Q20 instrument (PerkinElmer, Inc., Waltham, MA, USA). The glass transition temperatures (*T*_g_) were recorded in the second heating run at 10 °C/min in an N_2_ atmosphere, with an instrument temperature range of −60 to 200 °C. Stress–strain experiments were performed using a CMT6104 instrument (NSS Laboratory Equipment Co., Ltd., Shenzhen, China) with specimens prepared on a HAAKE Mini Jet II system (Thermo Scientific, Inc., USA). UV-visible light transmittance was measured on a CARY 300 spectrophotometer (Varian Medical Systems, Inc., CA, USA) within the range of 400–800 nm. The catalytic activity equation is: A = m/(n × t), where “m” is the mass of polypropylene (g), “n” is the molar quantity of catalyst (mol), and “t” is the polymerization reaction time (h).

### 2.3. Preparation of α-Diimine Nickel(II) Complexes

Synthesis of *N*,*N*-bis(2,6-diisopropylphenyl)-[5,7-bis(4-hydroxymethylphenyl)]-acenaphthylene-1,2-diimine ligand (**L1**).

The 2,6-diisopropylaniline (2.20 mL, 11.40 mmol) and 5,7-dibromoacenaphthylene-1,2-dione (1.30 g, 3.80 mmol) were dissolved in toluene. The *p*-toluene-sulfonic acid was added and the mixture refluxed for 6 h; the solvent was then evaporated and the residue was cooled to room temperature for column chromatography separation and purification of 0.50 g of compound **B1**. The compound **B1** (0.50 g, 0.76 mmol), *p*-hydroxymethyl phenylboronic acid (0.30 g, 1.90 mmol), and anhydrous potassium carbonate (0.80 g, 5.71 mmol) were poured into a 500 mL reaction flask, in which 4-(triphenylphosphine) palladium was used as a catalyst. In an argon atmosphere, tetrahydrofuran (30 mL), dried by calcium chloride, and distilled water (18 mL) were injected into the flask and heated for 10 h. After that, it was cooled to room temperature, extracted with dichloromethane, dried with anhydrous magnesium sulfate, pumped, concentrated, and then purified by column chromatography to obtain ligand **L1** with a yield of 64%. **L1**: MS(ESI): *m/z* 713(M+H) (Figure S1). ^1^H-NMR (400 MHz, CDCl_3_, δ, ppm): δ 7.38–7.33 (m, 8H), δ 6.97 (d, *J* = 8.0 Hz, 4H), δ 6.90 (d, *J* = 8.0 Hz, 4H), δ 6.82 (d, *J* = 8.0 Hz, 2H), δ 4.59 (s, 4H), δ 3.20–3.13 (m, 4H), δ 1.34 (d, *J* = 8.0 Hz, 12H), δ 1.11 (d, *J* = 8.0 Hz, 12H) (Figure S2). FT–IR (ν, cm^−1^): 2957, 2922, 2867, 1637 (C=N), 1581 (C=N), 1461, 1424, 1382, 1359, 1325, 1252, 1180, 1094, 1054, 1033, 972, 949, 854, 831, 794, 754, 699, 620, 545 (Figure S3). Elemental analysis C_50_H_52_N_2_O_2_ (712.9): Measured values (%): C, 84.53; H, 7.25; N, 4.59; O, 5.53. Theoretical values (%): C, 84.55; H, 7.22; N, 4.68; O, 5.49.

Synthesis of *N*,*N*-bis [2,6-diisopropylphenyl-4-(4-hydroxymethylphenyl)]-[5-(4-hydroxymethylphenyl)]-acenaphthylene-1,2-diimine ligand (**L2**).

A solution of 4-bromo-2,6-diisopropylaniline (2.60 mL, 13.50 mmol) reacted with bromoacenaphthylene-1,2-dione (2.20 g, 3.00 mmol) with *p*-toluene-sulfonic acid as the catalyst in toluene (30 mL) was used to prepare compound **B2**. Then, compound **B2** (0.20 g, 0.30 mmol), *p*-hydroxymethyl phenylboronic acid (0.165 g, 1.10 mmol), and anhydrous potassium carbonate (0.46 g, 3.30 mmol) were refluxed for 10 h in 30 mL tetrahydrofuran and 18 mL distilled water using a small amount of 4-(triphenylphosphine) palladium as the catalyst. The experimental procedures were similar to the ones above, and the yield of ligand **L2** was 67%. **L2**: MS(ESI): *m/z* 819(M+H) (Figure S4). ^1^H-NMR (400 MHz, CDCl_3_, δ, ppm): δ 8.00 (d, *J* = 8.0 Hz, 1H), δ 7.74 (d, *J* = 8.0 Hz, 4H), δ 7.51 (d, *J* = 16.0 Hz, 12H), δ 7.38–7.26 (m, 2H), δ 6.87–6.82 (m, 2H), δ 4.78 (s, 4H), δ 3.17–3.08 (m, 4H), δ 1.31 (d, *J* = 8.0 Hz, 12H), δ 1.09–1.05 (t, *J* = 8.0 Hz, 12H) (Figure S5). FT–IR (ν, cm^−1^): 3420, 3052, 3026, 2959, 2927, 2868, 2356, 1901, 1667 (C=N), 1640 (C=N), 1595, 1515, 1487, 1459, 1440, 1426, 1396, 1383, 1361, 1342, 1294, 1251, 1186, 1016, 938, 885, 782, 747 (Figure S6). Elemental analysis C_57_H_58_N_2_O_3_ (819.1): Measured values (%): C, 83.42; H, 7.10; N, 3.40; O, 5.35. Theoretical values (%): C, 83.52; H, 7.08; N, 3.42; O, 5.39.

Synthesis of *N*,*N*-bis(2,6-diisopropylphenyl)-[5,7-bis(4-hydroxymethylphenyl)]-acenaphthylene-1,2-diimine nickel dibromide (**C1**).

The ligand **L1** (1.01 mmol, 0.72 g) and (DME)NiBr_2_ (1.00 mmol, 0.31 g) were dissolved separately in 20 mL of CH_2_Cl_2_ and stirred for 8 h in a three-necked flask filled with argon gas. The CH_2_Cl_2_ solution of ligand **L1** was then added dropwise to that of (DME)NiBr_2_. The reaction mixture was refluxed at 40 °C and stirred for 48 h. After completion of the reaction, the upper CH_2_Cl_2_ was removed. The mixture was cleaned with n-hexane three times and dried by vacuum evaporation to obtain the brown-yellow powder **C1** with a yield of 86%. **C1**: FT–IR (ν, cm^−1^): 2962, 2927, 2868, 1619 (C=N), 1576 (C=N), 1463, 1422, 1384, 1364, 1328, 1232, 1183, 1100, 1058, 1043, 978, 936, 837, 798, 762, 700, 663, 614 (Figure S3). Elemental analysis C_50_H_52_Br_2_N_2_NiO_2_ (931.48): Measured values (%): C, 64.65; H, 5.52; N, 3.48; O, 6.30. Theoretical values (%): C, 64.47; H, 5.63; N, 3.44; O, 6.34.

Synthesis of *N*,*N*-bis [2,6-diisopropylphenyl-4-(4-hydroxymethylphenyl)]-[5-(4-hydroxymethylphenyl)]-acenaphthylene-1,2-diimine nickel dibromide (**C2**).

The experimental procedures and reactant molar ratios for the synthesis of **C2** were similar to those for **C1**, and the yield of red-brown **C2** was 73%. **C2**: FT–IR (ν, cm^−1^): 3434, 2963, 2865, 1910, 1946, 1649 (C=N), 1619 (C=N), 1596, 1579, 1516, 1486, 1461, 1421, 1384, 1339, 1300, 1261, 1180, 1093, 1072, 1014, 830, 808, 626 (Figure S6). Elemental analysis C_57_H_58_Br_2_N_2_NiO_3_ (1036.6): Measured values (%): C, 66.10; H, 5.62; N, 2.55; O, 4.74. Theoretical values (%): C, 65.98; H, 5.63; N, 2.70; O, 4.67.

Due to the presence of a nickel element, which can induce paramagnetic interference in NMR tests, complexes **C1** and **C2** were characterized using FT–IR and elemental analysis [21]. Comparing the IR spectra of ligands **L1** and **C1** (Figure S3), as well as those of **L2** and **C2** (Figure S6), it was noted that the C=N absorption peaks of ligand **L1** were attributed at 1637 cm^−1^ and 1581 cm^−1^, while those of complex **C1** were attributed at 1619 cm^−1^ and 1576 cm^−1^. Similarly, for ligand **L2**, the C=N absorption peaks were observed at 1667 cm^−1^ and 1640 cm^−1^, whereas for complex **C2**, they were detected at 1649 cm^−1^ and 1619 cm^−1^. In comparison to ligands **L1** (or **L2**), the C=N bond absorption peaks of complexes **C1** (or **C2**) were obviously blue-shifted, indicating coordination of the metal nickel with the ligand. Supported by the elemental analysis findings, it is confirmed that complexes **C1** and **C2** were synthesized.

### 2.4. Propylene Polymerization

The polymerization experiments took place in a 100 mL stainless steel vessel furnished with a pressure control system and a temperature-controlled magnetic stirring apparatus. Initially, the reactor was evacuated at 100 °C for two hours to eliminate moisture, and then cooled to room temperature under an argon atmosphere before being purged twice with argon and once with propylene. Then, the solvent, cocatalyst, and catalyst were injected, and the polymerization experiment was carried out at a controlled temperature and pressure. After reaching the reaction time, the propylene supply was cut off, and the polymerization experiment was stopped. The resultant mixture was quenched by the addition of a 10 vol% HCl/C_2_H_5_OH solution and stirred for at least 6 h. The polypropylene was washed alternately with C_2_H_5_OH and H_2_O and dried in a vacuum oven at 60 °C for 8 h.

## 3. Results and Discussion

### 3.1. Synthesis of α-Diimine Ligands and Complexes **C1** and **C2**

To study the effects of bulky substituents on the ligand’s *N*-aryl group and acenaphthequinone-backbone on improving the catalytic activity and molecular weight of the polymer, we designed and synthesized two α-diimine nickel complexes, **C1** and **C2**.

**Scheme 2:**
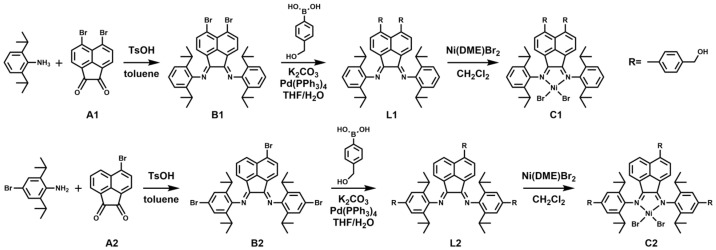
Synthesis process of complexes **C1** and **C2**.

The synthetic procedures of α-diimine nickel(II) complexes are shown in Scheme 2. These ligands and complexes were characterized. The structure of α-diimine nickel complex **C1** contained two hydroxymethyl phenyl substituents on the side of the acenaphthequinone-backbone, and there were three hydroxymethyl phenyl substituents on the para-site structure of *N*-aryl and acenaphthequinone-backbone of complex **C2**. The hydroxymethyl phenyl substituent plays an important role in improving catalytic behaviors.

### 3.2. Propylene Polymerization with α-Diimine Nickel (II) Complexes **C1** and **C2**

Using complexes **C1** and **C2** as catalysts for propylene polymerization, we observed that the resultant polypropylenes were transparent elastomers. The results are shown in Table 1 and Table 2. The results show that catalysts **C1** and **C2** had the maximum catalytic activity at 5.40 × 10^5^ g PP/mol Ni·h and 4.53 × 10^5^ g PP/mol Ni·h, respectively, and the activity still maintained above 10^5^ g PP/mol Ni·h at 50 °C, indicating the good thermal stability of these catalysts. Compared with the α-diimine catalysts reported in the literature, these two catalysts showed good activity in catalytic propylene polymerization in toluene, a commonly used polymerization solvent in industry. This should be related to the hydroxymethyl phenyl substituents in the ligand structure. The hydroxymethylphenyl on the ligand is not a very greatly bulky group, but the hydroxy groups may react with the cocatalyst to form bulky substituents, and also react with the cocatalyst anion of the active center ion pair to increase the distance between cation species and anion, making propylene monomer insertion easier and improving thermal stability and catalytic activity [44,45,46]. Meanwhile, the catalytic activity of catalyst **C1** was higher than that of **C2** under the same polymerization conditions, probably due to the absence of large steric hindrance substituents on the *o*-*N*-aryl of the **C1** ligand, which was more conducive to the coordination and insertion of propylene.

Furthermore, the effects of the cocatalyst type, Al/Ni molar ratio, polymerization temperature, and solvent on catalytic activity were investigated. The MAO, AlEt_2_Cl and AlEt_3_ were used as cocatalysts for polymerization, respectively. The activity of the DEAC system was 3.67 × 10^5^ g PP/mol Ni·h (Entry 2 in Table 1), while the catalytic activity of the MAO system was 0.93 × 10^5^ g PP/mol Ni·h, and only trace polymer was obtained in the AlEt_3_ system under the same polymerization conditions. This could result from the different activation abilities of the cocatalyst to the catalyst. The low-cost AlEt_2_Cl is a more efficient alkylaluminum cocatalyst because of its more suitable Lewis acidity and steric bulk for the α-diimine catalyst [47,48]. **C1**/**C2** activity was highest when the Al/Ni molar ratio was 1200. This is because too much cocatalyst may lead to an increase of chain-transfer reaction to the cocatalyst, resulting in a decrease in activity.

The experimental results show that polymerization temperature significantly affects activity. At lower polymerization temperatures, such as between −20 °C and 30 °C, the **C1**/**C2** activity was significantly higher than that at higher polymerization temperatures. They exhibited the highest catalytic activity at 0 °C. When the polymerization temperature was increased above 50 °C, the decrease in activity was attributed to increased *N*-aryl rotations, leading to C-H activation between the metal center and the ortho-substituent of *N*-aryl and the formation of an inactive cyclized metal complex [49,50]. In addition, the solubility of propylene gas in toluene decreases with an increase in temperature, resulting in a decrease in monomer concentration around the active center and thus in the rate of chain growth. The elevated temperature also accelerated the rate of chain transfer.

The pressure of propylene still has a certain effect on activity. The activity of **C1** is 2.33 × 10^5^ g PP/mol Ni·h at 0.1 MPa, and when the pressure was increased to 0.5 MPa, the activity increased to 3.67 × 10^5^ g PP/mol Ni·h. However, the catalytic activity decreased to 2.87 × 10^5^ g PP/mol Ni·h when the pressure further increased to 0.7 MPa. According to the literature [38], this phenomenon may be related to the fact that the higher monomer concentration is close to the active center of the catalyst, which could increase the temperature in the polymerization and decay the active centers quickly. We compared the polymerization of catalyst **C1** in toluene and n-hexane, two commonly used industrial solvents. From the comparative data of Entry 2&9 (3.67 × 10^5^ gPP/molNi·h & 3.10×10^5^ g PP/mol Ni·h) and Entry 6&10 (1.93 × 10^5^ g PP/mol Ni·h & 1.60 × 10^5^ g PP/mol Ni·h), the activities of **C1** in n-hexane solvent were slightly lower than in the toluene system, which was related to the lower polarity of n-hexane and the lower polarization degree of the ion pairs in the active center. The relative closeness of the cation metal center to the cocatalyst anion is not conducive to an insertion growth reaction of the monomer. Nevertheless, the catalyst exhibited good activity in n-hexane, which has not been reported in the literature.

### 3.3. Molecular Weight and Chain Structure of Polypropylene

Considering the material properties, especially the mechanical properties, it is an urgent problem to improve the molecular weight of polypropylene obtained by α-diimine. The literature states that the polypropylenes obtained from α-diimine nickel catalysts above room temperature (25 °C) had the highest *M*_w_ of 717 kg/mol [51]. At 30 °C, we obtained polypropylene with an *M*_w_ of 925 kg/mol, offered by **C2**. Moreover, at 0 °C, polypropylene prepared by **C1** had the highest molecular weight (*M*_w_ = 1101 kg/mol), a value that is commonly 50–600 kg/mol in the literature [15,35,36,38,42,47].

It is widely accepted that the structure of the catalyst is an essential factor affecting the molecular weight of the polymer. The steric hindrance of substituent on the ligand has obvious effects on monomer insertion, chain growth, and the chain-transfer reaction. Therefore, the design of the catalyst structure should consider not only the increase in polymerization activity but also the impact on polymer molecular weight. In the above study of this work, the two catalysts **C1** and **C2** have shown good catalytic activity. We next investigated the molecular weights of polypropylenes prepared with these two catalysts. The GPC data show that the average molecular weights (*M*_w_) of polypropylenes obtained from catalysts **C1** and **C2** at 30 °C were 736 kg/mol and 925 kg/mol, and the *M*_w_ of the polypropylene was still above 400 kg/mol at 50 °C, which are higher than those of reported in literature. The molecular weight of the polypropylene was significantly improved using our provided catalysts with large steric hindrance substituent structures, and the high molecular weight of the polymer will further improve the mechanical properties of the material.

**Figure 1:**
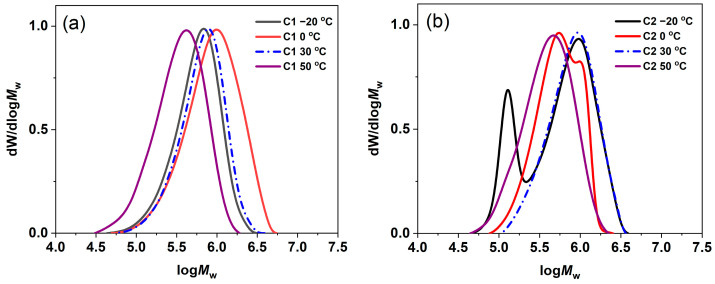
GPC curves of resultant polypropylenes: (**a**) Entries 2, 4, 5, 6; (**b**) Entries 12, 14, 15, 16.

In a comparison of the molecular weights of polypropylenes synthesized with these two catalysts, the molecular weights of polypropylenes prepared by catalyst **C2** were higher than those prepared by catalyst **C1** above 30 °C (Figure 1). This is due to the fact that the larger steric hindrance substituents of catalyst **C2**, both on acenaphthequinone-backbone and *N*-aryl, inhibit the chain-transfer reaction, resulting in longer polymer chains and higher molecular weights. Furthermore, there are some unique results. The *M*_w_ of polypropylene obtained by catalyst **C1** at 0 °C was as high as 1101 kg/mol, which is the highest molecular weight reported in the existing literature. Note also that Table 2 and Figure 1b indicate that polypropylenes with bimodal molecular weight distribution were prepared by catalyst **C2** at the polymerization temperature of −20 °C and 0 °C. This could be explained by the asymmetric structure of **C2**. The difference between the two sides of the active center results in a significant difference in the monomer insertion reactions on either side of the active center, thereby forming different polymer molecular weights [40,52]. However, with an elevated polymerization temperature, the selectivity of the monomer insertion direction decreased, and the GPC curves became a single peak.

We further investigated the effect of the polymerization condition on the molecular weight of the polypropylene. When the polymerization temperature was −20 °C, the molecular weight of the polypropylene was not high due to the low chain growth. As the polymerization temperature rose, the rate of chain growth and monomer insertion accelerated, leading to an increase in the molecular weight of the polymer. However, as the polymerization temperature was further elevated to 50 °C, the molecular weight of the polypropylene decreased. This phenomenon is attributed to the increase in chain-transfer reactions and the corresponding decrease in the solubility of propylene monomers in toluene. The polypropylene had the highest molecular weight when the Al/Ni molar ratio was 1200. After a further increase in the Al/Ni molar ratio, the molecular weight of the polymer decreased because too many cocatalysts acted as chain transfer agents (Figure S7). When the polymerization pressure was increased from 0.5 MPa to 0.7 MPa, the *M*_w_ of **C1** increased from 736 kg/mol to 808 kg/mol (Figure S8).

It is evident from the literature that polypropylenes synthesized by α-diimine catalysts exhibit branches and are very similar to ethylene–propylene copolymers with adjacent methylene-sequence groups, which are associated with the “chain walking” and “chain straightening” that occur after *β*-H elimination. Thus, the microstructures of the polypropylene samples were characterized, and, according to [53], the branching density, [CH_3_]/[CH_2_] unit ratio, and 1,3-enchainment content can be presented by analyzing ^1^H NMR (Figure S9).

As illustrated in Table 3, the resulting polypropylenes exhibited high branching densities (234–280/1000C), although these were lower than the theoretical value (333/1000C). This is attributed to the branching density being reduced by the presence of the methylene sequence generated from the 1,3-enchainment or “chain straightening” [32,33]. Compared with the values obtained at similar polymerization temperatures in the literature, the 1,3-enchainment contents of the resultant polypropylenes prepared by **C1** and **C2** were still relatively low (3.57–16.96%). This indicates that the structure of the large steric hindrance α-diimine nickel catalyst can effectively inhibit the 1,3-insertion reaction that occurs during polymerization.

The results show that ligand structure and polymerization temperature can effectively adjust the branching density and 1,3-enchainment content. The complex **C2**, featuring three large steric hindrance hydroxymethyl phenyl substituents, results in a significantly lower percentage of 1,3-insertion in the polypropylene structure compared to **C1**. This indicates that it is feasible to suppress the 2,1- and 1,3-insertion processes through modifications to the ligand structure in the α-diimine nickel system [34,40]. Higher 1,3-enchainment contents were observed at higher polymerization temperatures, which may be caused by various factors, including the activation energy of chain walking and solution viscosity, etc. [34].

**Figure 2:**
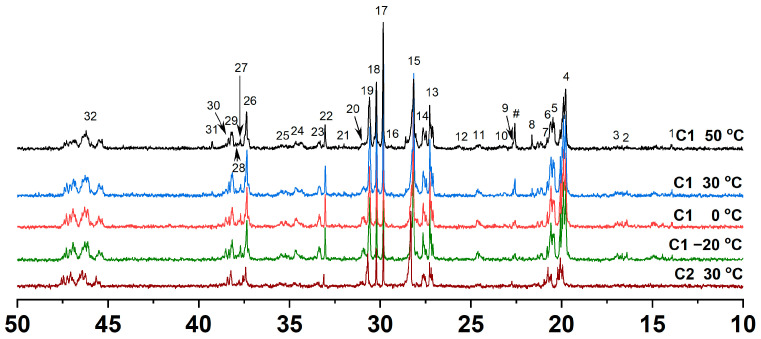
^13^C NMR spectra of polypropylenes (Entries 2, 4, 5, 6, 12).

To further investigate the branches of polypropylene, we employed ^13^C-NMR to characterize polypropylene samples (Figure 2) and analyzed these results in Table S1 and Figure S10 [54]. Initially, it was observed that the chain structure of polypropylene has a clear methylene sequence generated from the 1,3-enchainment ([EEE], δ~29.84 ppm). In addition, alkyl side-chains resulting from “chain walking” have been observed. These include the presence of iso-butyl (*i*Bu), 2-methyl hexyl (2MH), and long chains (L, more than 6 carbon atoms), confirmed by the presence at δ~23.0–24.0 ppm, δ~22.68 ppm, and δ~13.97 ppm, respectively. However, some common branched chains of ethyl, propyl, and pentyl branches in polyethylene were not observed [42]. The sequence structure was calculated, and the results are presented in Table 4. With the increase in temperature from −20 °C to 50 °C, the [EEE] content in the polypropylenes prepared by catalyst **C1** increased from 6.4% to 13.3%. The change trend is consistent with the 1,3-enchainment content results of the ^1^H-NMR calculation. As well as the structural changes in the main chain, the type and number of branches also increased with elevated temperature. Below 0 °C, the branches of iso-butyl, 2-methyl hexyl, and long chains can hardly be observed. However, the contents of [ELE], [E*i*BuE], and [E2MHE] increased to 0.8%, 1.3%, and 1.6% at 50 °C. This indicates that the elevated temperature promotes the occurrence of chain walking and leads to an increase in the type and number of branches on the polymer chain segments. The average sequence length of the E-unit was calculated from *n*_E_ = ([EEE] + ([PEE] + [EEP]) + [PEP])/(1/2([PEE] + [EEP]) + [PEP]) [53]. The values of *n*_E_ = 2.24, 2.32, 2.77, and 3.47 were concluded when elevating the temperature from −20 °C to 50 °C. Moreover, it was also observed that the polypropylene synthesized by catalyst **C2**, with its larger steric hindrance, had fewer branches than **C1** at the same temperature.

**Figure 3:**
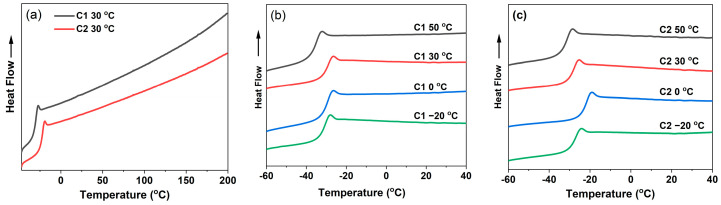
DSC curves of polypropylenes: (**a**) Entries 2, 12; (**b**) Entries 2, 4, 5, 6; (**c**) Entries 12, 14, 15, 16.

The presence of branches and 1,3-enchainment on the polypropylenes further led to the irregularity of the chain structure, and the polymer chains could not be regularized for crystallization, as evidenced by the DSC curves (Figure 3). These polypropylenes did not exhibit melting temperatures *T*_m_ within the range of −50 °C to 200 °C (Figure 3a). The glass transition temperature (*T*_g_) was only observed between −36 °C and −23 °C, which was considerably lower than that of isotactic polypropylene (−10 to 5 °C). The *T*_g_ of polypropylene prepared by catalyst **C2** was consistently higher than that obtained from catalyst **C1** under the same conditions. In Figure 3b,c, we can see that the *T*_g_ of polypropylene increased when the polymerization temperature decreased from 50 °C to 0 °C, except for −20 °C. We believe that this change trend is closely related to the chain structure and molecular weight of the polymer. However, the molecular weight of polypropylene obtained at −20 °C is lower than that obtained at 0 °C. With the decrease in the molecular weight of the polymer, the proportion of the chain segments at the end of the chain increased, resulting in a decrease in the glass transition temperature.

### 3.4. Properties of Polypropylene

**Figure 4:**
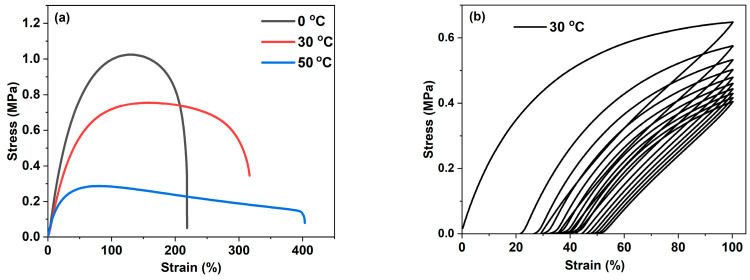
(**a**) Stress–strain curves for polypropylenes (Entries 2, 5, 6); (**b**) Plots of hysteresis experiments of 10 cycles at a strain of 100% for polypropylene (Entry 2).

The mechanical properties of polyolefin material prepared by late transition metal catalysts are very worthy of research. The amorphous polypropylene with higher molecular weight prepared by our catalysts exhibits improved elastic properties (Figure S11). Herein, the mechanical and elastic properties of the polypropylene samples were studied (Figure 4, Table S2).

In the stress–strain curves of Figure 4a, the polypropylene samples demonstrated low tensile strength, ranging from 0.3 to 1.0 MPa, but high elongation at break from 218% to 403%. There is no obvious yield point on the stress–strain curves, showing the soft and tough characteristics of the elastomer [55]. As the polymerization temperature increased from 0 °C to 50 °C, the tensile strength of polypropylene decreased from 1.0 MPa to 0.3 MPa, which is related to the decrease in the molecular weight of the polymer. Meanwhile, the elevated temperature resulted in an increase in the type and number of branches on the chain structure, leading to more entanglement between the polymer chains and an increase in elongation at break. To further examine the polypropylene’s capacity to revert to its original state upon tension release, a cyclic stress–strain test was performed to assess its elasticity. As shown in Figure 4b, the polypropylene sample was subjected to 10 repeated stress–strain cycles at 100% strain. After each cycle, it failed to revert completely to its initial state, with the first cycle resulting in the most significant deformation, suggesting both elastic and plastic deformation during the tension process. Even after 10 tension cycles, the strain recovery (SR) value of polypropylene remained at 50%, demonstrating its good elastic recovery capabilities.

**Figure 5:**
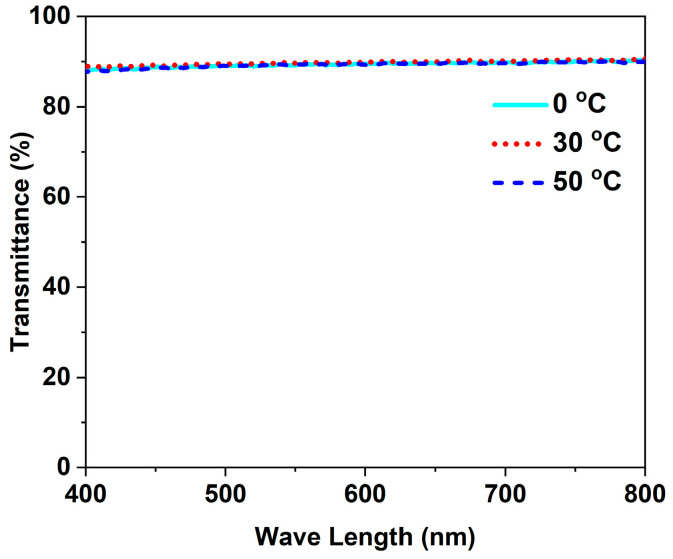
UV-vis transmittance curves for polypropylenes (Entries 2, 5, 6).

Due to the irregular molecular chain structure, the resulting polypropylene cannot crystallize and exhibit transparency. The light transmittance of the polypropylene prepared by **C1** was measured across the UV-visible light spectrum, ranging from 400 to 800 nm. As Figure 5 shows, polypropylenes at different polymerization temperatures demonstrated a high visible light transmittance of approximately 90%.

## 4. Conclusions

In summary, two α-diimine nickel catalysts with multiple hydroxymethyl phenyl substituents were synthesized and used for propylene homopolymerization. Due to the presence of large steric hindrance substituents, the catalysts exhibited high activity and good thermal stability in toluene, enabling the preparation of high-molecular-weight homopolypropylene (up to 1101 kg/mol). Even at 50 °C, the catalytic activities were above 10^5^ g PP/mol Ni·h, and the *M*_w_ of polypropylene reached 480 kg/mol. The polypropylenes prepared using asymmetric complex **C2** exhibited bimodal molecular weight distribution at −20 and 0 °C, reflecting their unique catalytic properties. The resulting polypropylenes displayed methylene-sequence and branches in the chain structure, with high branching density (234~280/1000C), low 1,3-enchainment content (3.57~16.96%), and low *T*_g_ (−36~−23 °C). The low tensile strength and high elongation at break confirmed the elastomeric properties of the propylene. The structure of the catalyst and polymerization conditions significantly influence polymerization activity, enabling the regulation of polypropylene properties such as molecular weight, branching density, 1,3-insertion content, and stress–strain behavior. This suggests that modification of the catalyst structure is an effective means of controlling catalyst properties and polymer molecular weight. The exploration of improving the properties of elastomer further by adding comonomers, such as ethylene, and toughening isotactic polypropylene by in situ polymerization is in progress.

## Figures and Tables

**Table 1:** The results of propylene polymerization catalyzed by complex **C1** ^a^.

Entry	*T* °C	n(Al)/ n(Ni)	*P* MPa	Yield g	Act. ^b^	*M*_n_^c^ kg/mol	*M*_w_^c^ kg/mol	PDI ^c^	*T*_g_ °C
1	30	900	0.5	0.97	3.23	397	654	1.6	n.d.
2	30	1200	0.5	1.10	3.67	373	736	1.9	−33.2
3	30	1500	0.5	0.75	2.50	379	582	1.5	n.d.
4	−20	1200	0.5	0.97	3.23	401	661	1.6	−31.7
5	0	1200	0.5	1.62	5.40	548	1101	2.0	−30.3
6	50	1200	0.5	0.58	1.93	215	407	1.9	−35.8
7	60	1200	0.5	0.15	0.50	n.d.	n.d.	n.d.	n.d.
8	30	1200	0.7	0.86	2.87	425	808	1.9	n.d.
9 ^d^	30	1200	0.5	0.93	3.10	391	657	1.7	n.d.
10 ^d^	50	1200	0.5	0.48	1.60	131	250	1.9	n.d.

^a^ Condition: n(Ni) = 3 μmol, AlEt_2_Cl for Cocatalyst, V(toluene) = 50 mL; n.d. = not determined; ^b^ Activity = 10^5^ gPP/molNi·h; ^c^ Determined by GPC; ^d^ V(n-hexane) = 50 mL.

**Table 2:** The results of propylene polymerization catalyzed by complex **C2** ^a^.

Entry	*T* °C	n(Al)/ n(Ni)	*P* MPa	Yield g	Act. ^b^	*M*_n_^c^ kg/mol	*M*_w_^c^ kg/mol	PDI ^c^	*T*_g_ °C
11	30	900	0.5	0.67	2.23	493	882	1.8	n.d.
12	30	1200	0.5	1.02	3.40	562	925	1.6	−28.9
13	30	1500	0.5	0.70	2.33	483	798	1.7	n.d.
14	−20	1200	0.5	0.53	1.77	116/667	127/978	2.6	−30.0
15	0	1200	0.5	1.36	4.53	404	616	1.5	−22.7
16	50	1200	0.5	0.50	1.67	268	480	1.8	−32.3
17	60	1200	0.5	0.13	0.43	n.d.	n.d.	n.d.	n.d.

^a^ Condition: n(Ni) = 3 μmol, AlEt_2_Cl for Cocatalyst, V(toluene) = 50 mL; ^b^ Activity = 10^5^ gPP/molNi·h; ^c^ Determined by GPC; n.d. = not determined.

**Table 3:** ^1^H-NMR data for polypropylenes.

Entry	Catalyst	*T* °C	*P* MPa	B ^a^	[CH_3_]/[CH_2_] ^b^	%1, 3 ^c^
5	C1	0	0.5	257	0.86	5.15
2	C1	30	0.5	249	0.73	10.98
6	C1	50	0.5	234	0.62	16.96
8	C1	30	0.7	242	0.68	13.56
15	C2	0	0.5	280	0.90	3.57
12	C2	30	0.5	254	0.74	10.48

^a^ B = branches per 1000 carbons, branching numbers were determined using ^1^H-NMR spectroscopy; ^b^ Determined by ^1^H-NMR spectroscopy; ^c^ Determined by the equation: %1,3-insertions = [(1 − R)/(1 + 2R)] × 100, where R = [CH_3_]/[CH_2_].

**Table 4:** Microstructure analysis of polypropylenes.

Sequences	Molar Content (%)				
	C1/−20 °C	C1/0 °C	C1/30 °C	C1/50 °C	C2/30 °C
[PPP]	40.9	41.7	41.2	41.5	42.0
[PPE+EPP]	9.8	8.5	10.2	7.3	9.8
[EPE]	4.9	4.7	4.7	5.6	5.0
[EEE]	6.4	6.9	9.4	13.3	9.2
[PEE+EEP]	13.8	13.6	12.5	9.0	12.1
[PEP]	3.8	3.6	2.6	2.7	2.4
[PP*P]	1.9	2.2	0.8	0.0	1.2
[PPP*]	3.1	3.3	2.4	0.8	2.6
[PP*PP]	5.2	5.5	4.5	3.7	4.8
[P*P*PP]	3.0	3.2	3.4	4.4	3.3
1,4-B_1_betE	2.6	2.1	1.8	1.9	1.7
1,6-β’B_1_	4.6	4.7	5.2	6.1	5.4
[ELE]	0.0	0.0	0.4	0.8	0.3
[E*i*BuE]	0.0	0.0	0.0	1.3	0.0
[E2MHE]	0.0	0.0	0.9	1.6	0.2

## Data Availability

The original contributions presented in the study are included in the article/Supplementary Material and further inquiries can be directed to the corresponding authors.

## Supplementary Materials

The following supporting information can be downloaded at: https://www.mdpi.com/article/10.3390/polym16162376/s1, Figure S1: The MS(ESI) spectrum of **L1**; Figure S2: The ^1^H NMR spectrum of **L1**; Figure S3: FT-IR spectra of **L1** and corresponding complex **C1**; Figure S4: The MS(ESI) spectrum of L2; Figure S5: The ^1^H NMR spectrum of **L2**; Figure S6: FT-IR spectra of **L2** and corresponding complex **C2**; Figure S7: GPC curves of polypropylenes prepared by **C1** and **C2**; Figure S8: GPC curves of polypropylenes prepared by **C1**; Figure S9: The ^1^H-NMR spectra of polypropylenes; Figure S10: Branched units of polypropylenes; Table S1: Nuclear magnetic carbon spectroscopy of polypropylene; Figure S11: The stretching of polypropylene; Table S2: Mechanical properties of polypropylenes prepared by **C1**.
