# Supplementary material for: Synthesis of High-Molecular-Weight Polypropylene Elastomer by Propylene Polymerization Using α-Diimine Nickel Catalysts
Source: Polymers (Basel). 2024 Aug 22;16(16):2376. doi: 10.3390/polym16162376 (PMC11359258; doi:10.3390/polym16162376)
Supplement: Supplementary file 1 [file polymers-16-02376-s001.zip › polymers-3105011-supplementary.pdf]

Supplementary Information

Article

Synthesis of High-Molecular-Weight Polypropylene Elastomer by Propylene Polymerization Using α-Diimine Nickel Catalysts

Lujie Gao [1,†], Hegang Ren [2,†], Yanhui Hou [3,*], Linlin Ye [1], Hao Meng [3], Binyuan Liu [1] and Min Yang [1,*]

[1] Hebei Key Laboratory of Functional Polymers, Institute of Polymer Science and Engineering, Hebei University of Technology, Tianjin 300401, China; gaolujie0511@163.com (L.G.); yell_23@163.com (L.Y.); byliu@hebut.edu.cn (B.L.)

[2] School of Material Science and Engineering, Guangdong University of Petrochemical Technology, Maoming 525000, China; rhg@gdupt.edu.cn

[3] State Key Laboratory of Separation Membranes and Membrane Processes, School of Material Science and Engineering, Tiangong University, Tianjin 300160, China; menghao@163.com

* Correspondence: houyanhui@tiangong.edu.cn (Y.H.); minyang@hebut.edu.cn (M.Y.); Tel.: +86-22-6020-4305 (M.Y.)

† These authors contributed equally to this work.

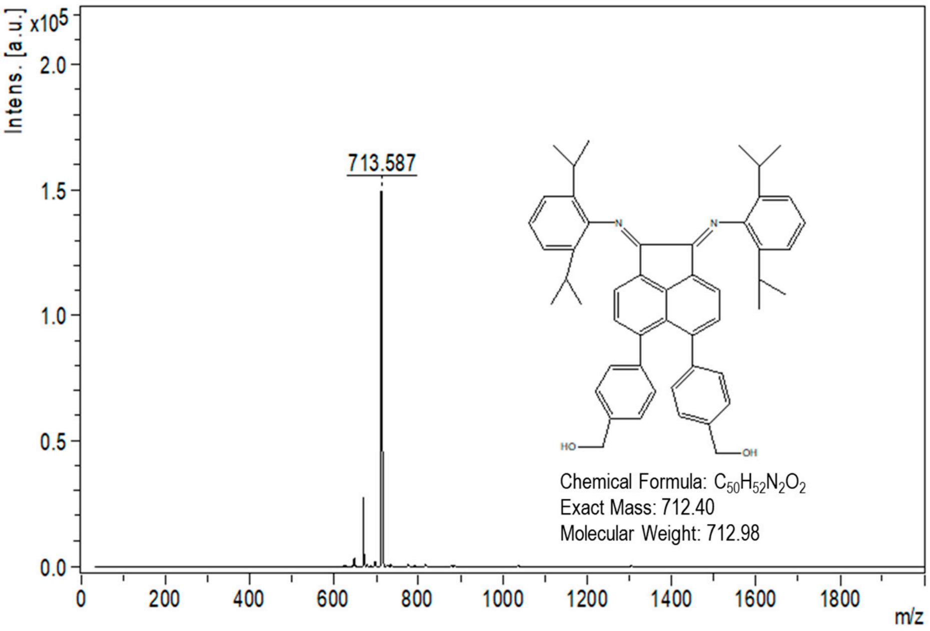

Figure S1 The MS(ESI) spectrum of **L1**

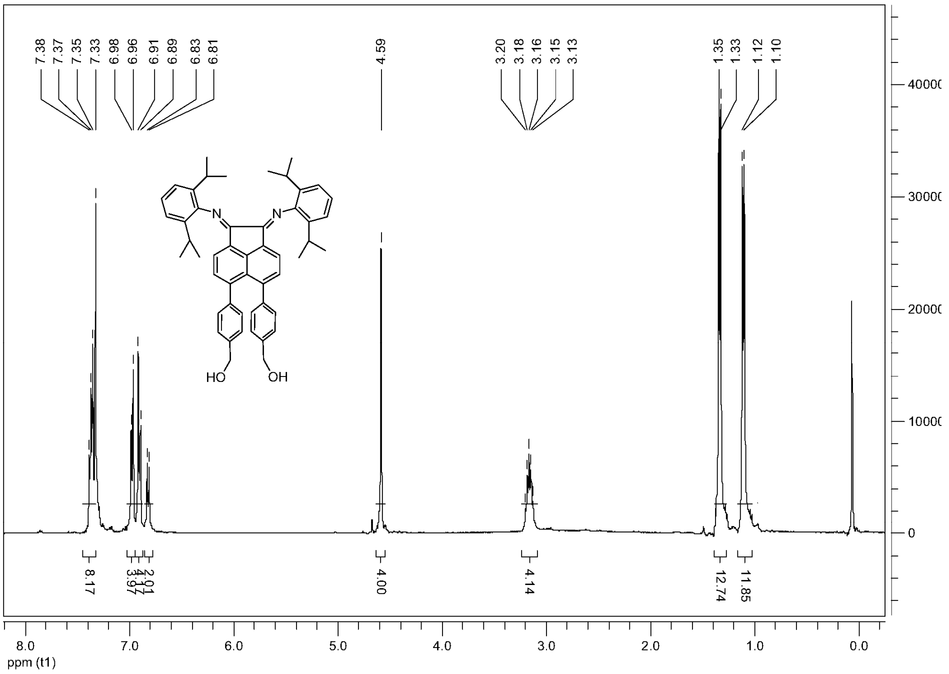

Figure S2 The ^{1}H NMR spectrum of **L1**

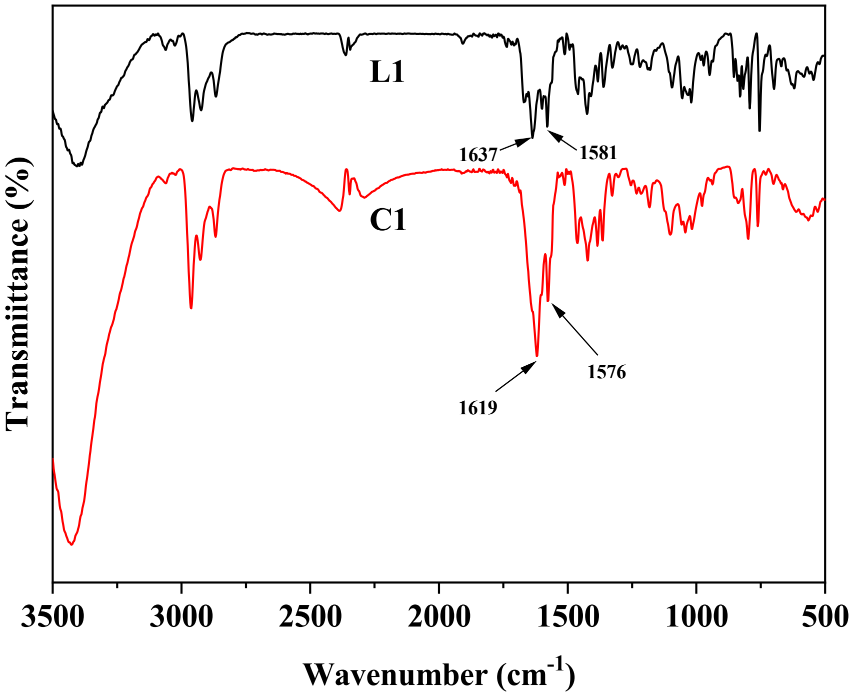

Figure S3 FT-IR spectra of L1 and corresponding complex C1

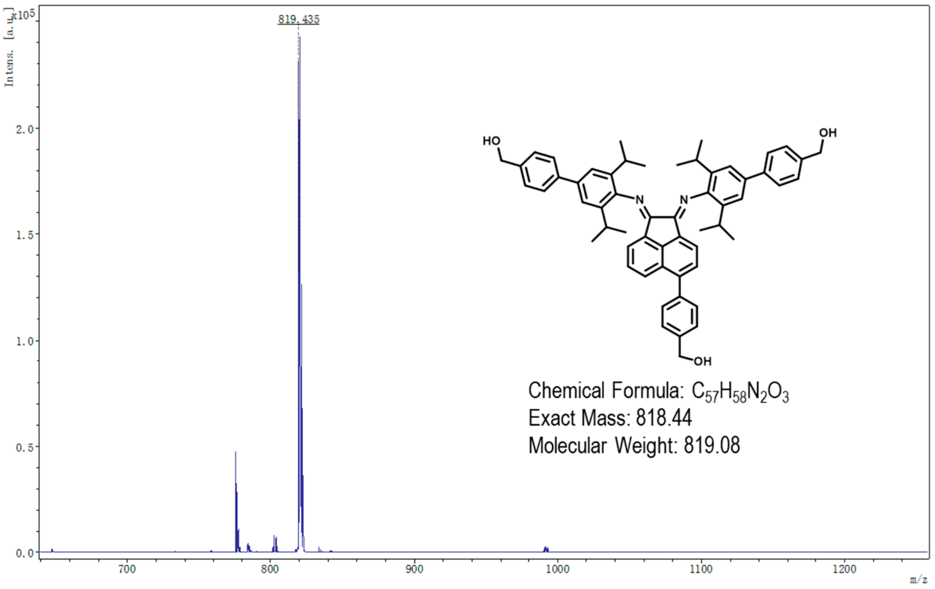

Figure S4 The MS(ESI) spectrum of **L2**

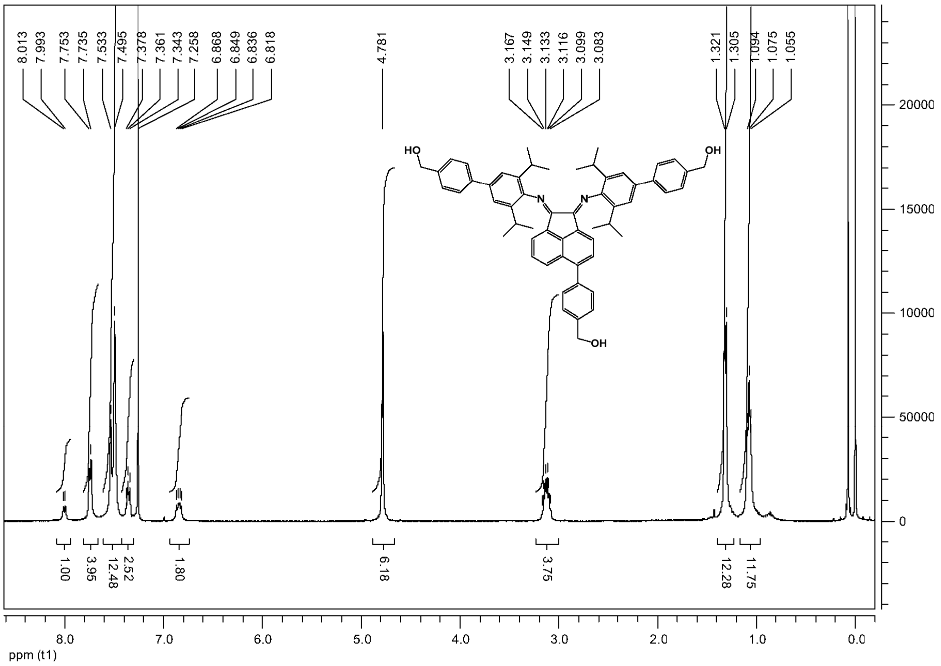

Figure S5 The ^{1}H NMR spectrum of **L2**

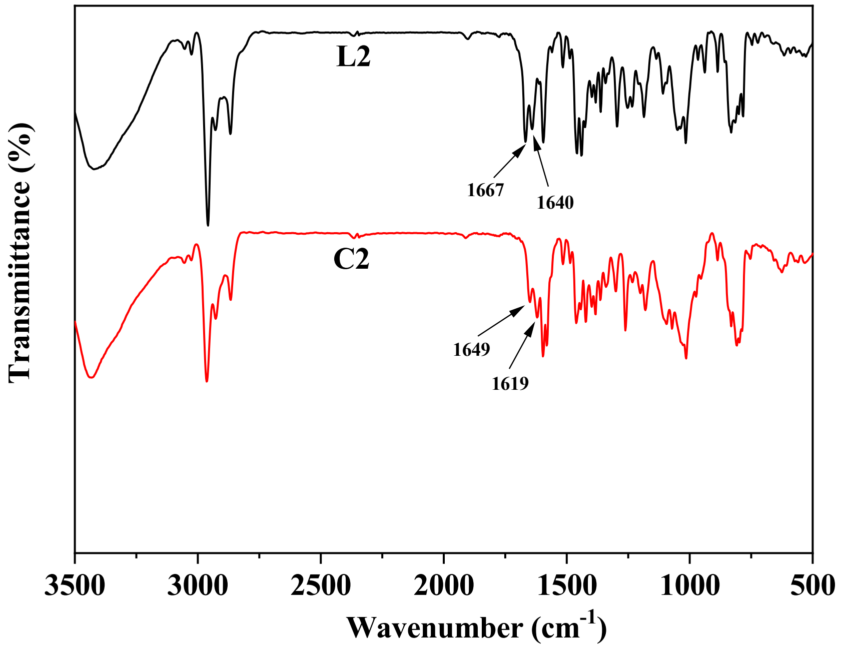

Figure S6 FT-IR spectra of **L2** and corresponding complex **C2**

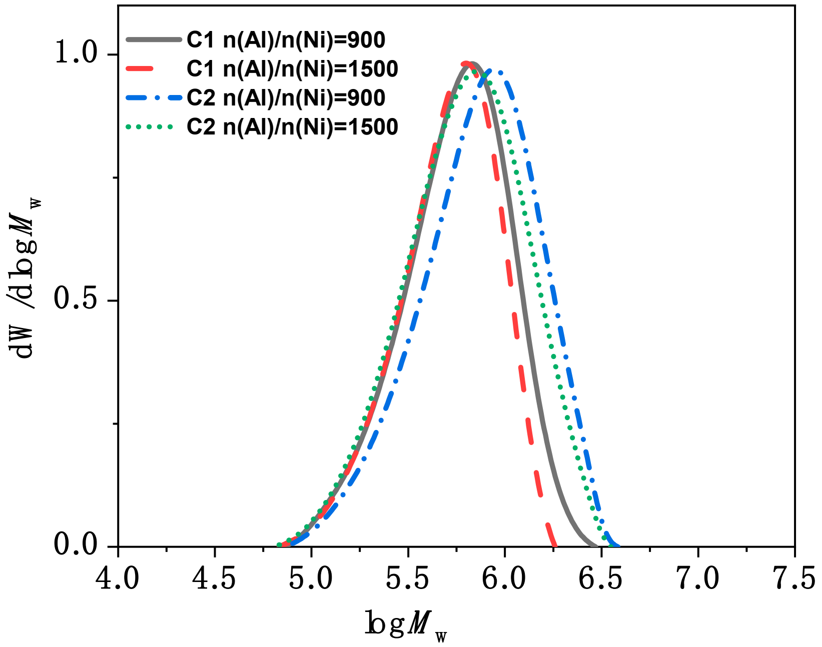

Figure S7 GPC curves of polypropylenes prepared by **C1** and **C2** (Entries 1, 3 in Table 1, Entries 11, 13 in Table 2)

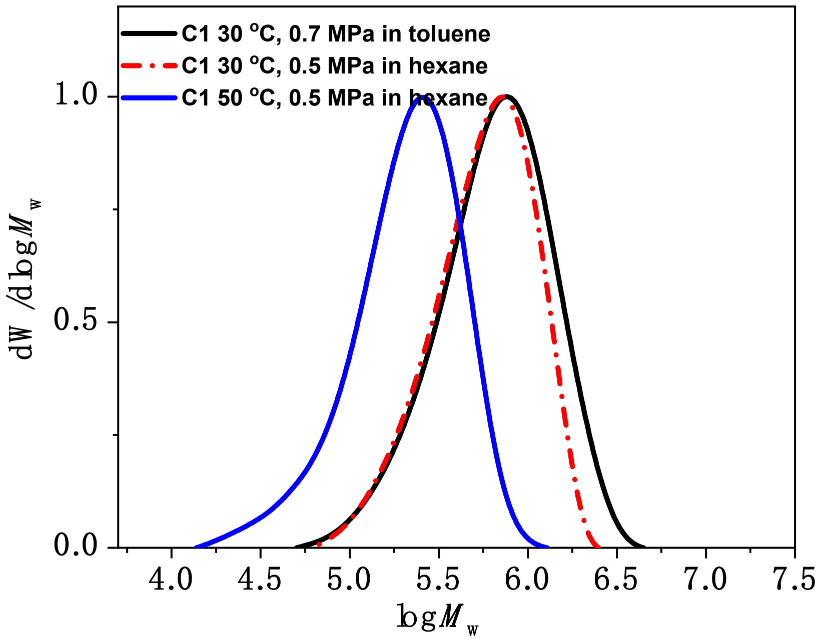

Figure S8 GPC curves of polypropylenes prepared by **C1** (Entries 8, 9, 10 in Table 1)

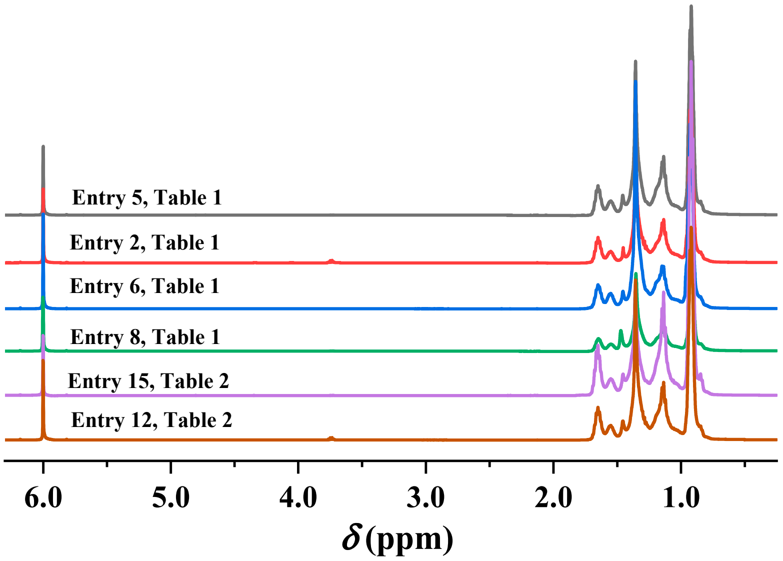

Figure S9 The ^{1}H-NMR spectra of polypropylenes

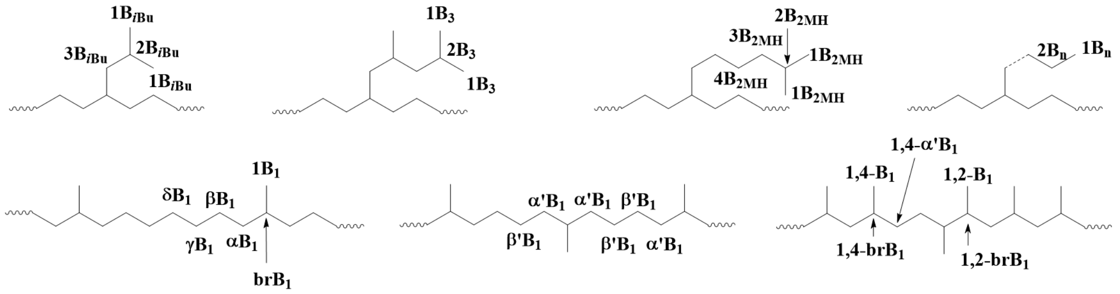

Figure S10 Branched units of polypropylenes

Table S1 Nuclear magnetic carbon spectroscopy of polypropylene

Peak Number	Chemical Shift Experiment (ppm)	Chemical Shift Calculation 1 (ppm)	Chemical Shift Calculation 2 (ppm)	Sequence	Carbon	
1	13.97	14.13	13.86	ELE	$1B_n$	
2	16.40	14.3-16.6	16.64	PP*P	$1B_1$	$P_{\alpha\gamma}$
3	16.68	16.72	17.13	PPP*	$1B_1$	$P_{\alpha\beta}$
	16.91	17.01				
4	19.6-20.2	19.5-20.2	20.61	PPPrr	$1B_1$	$P_{\beta\beta}$
	19.78	19.78		PPPPPmrrm		
	19.90	12.92		PPPPPmrrr		
	19.98					
	20.08	20.07		PPPPPrrrr	$1B_1$	$P_{\beta\beta}$
5	20.3-20.5					
	20.42	20.43	20.61	PPPPPmrmr	$1B_1$	$P_{\beta\beta}$

	20.48	20.57	20.12	EPP+PPE	$1B_1$	$P_{\beta\delta}$
				P*PP	$1,4\text{-}B_1$	$P_{\beta\gamma}$
6	20.60	20.67	20.61	PPPPPmrmm+rmrr	$1B_1$	$P_{\beta\beta}$
7	20.81	20.81	20.61	PPPPPmmrr	$1B_1$	$P_{\beta\beta}$
8	21.0-21.7	20.95-22.0	20.61	PPPmm	$1B_1$	$P_{\beta\beta}$
#	22.58					
9	22.68	22.82	22.13	E2MHE	$1B_{2MH}$	
10	23.0-24.0	23.0-24.0	22.62	E*i*BuE	$1B_{iBu}$	
			22.65	ELE	$2B_n$	
11	24.4-24.8	24.0-25.0	24.58	PEP	$\beta'B_1$	$S_{\beta\beta}$
12	25.5-25.8					
	25.64	25.65	25.92	E*i*BuE	$2B_{iBu}$	
13	27.0-27.4	27.10-27.55	27.27	E2MHE	$4B_{2MH}$	
	27.27		27.27	PEE+EEP	βB_1	$S_{\beta\delta}$
14	27.4-27.8	27.51-27.81				
	27.47		27.51, 27.9			$S_{\beta\gamma}$
	27.64		27.77			
15	27.8-28.7					
	28.03	29.97	27.99	E2MHE	$4B_{2MH}$	
	28.16		28.4			$T_{\beta\beta}$(mr+rr)
	28.23					
	28.59		28.7			$T_{\beta\beta}$(mm)
16	29.38	29.58	29.71	ELE	$4B_n$	
17	29.84	30.00	29.96	EEE	$\delta B_1,\delta B_n$	$S_{\delta\delta}$
18	30.21	30.35	30.21	EEEP	γB_1	$S_{\gamma\delta}$
19	30.4-30.8	30.4-30.6	30.21	ELE	$\gamma B_n+(n-2)B_n$	
				E2MHE	γB_{2MH}	
				E*i*BuE	γB_{iBu}	
			30.45	P*PP		$T_{\beta\gamma}$
	30.58	30.44	30.8	EPP+PPE	$1,4\text{-}brB_1$	$T_{\beta\delta}$
	30.62	30.52			brB_1	
20	30.8-31.1	30.70	30.46	PEEP	$1,4\text{-}\alpha'B_1$	$S_{\beta\alpha\beta\delta}$
		30.79	31.53	PP*P	$3B_n$	
21	31.99	32.12	32.40	ELE	$3B_n$	
22	33.04	33.10	32.52	EPE	brB_1	$T_{\delta\delta}$
23	33.2-33.5	33.38	32.52	EP*PE+EP*PE	$1,4\text{-}brB_1$(between E)	$T_{\gamma\delta}$
						$T_{\gamma\gamma}$
24	34.2-34.8					
		34.29	34.47	P*P*PP+P*P*PP	$1,4\text{-}\alpha'B_1$	$S_{\gamma\alpha\beta\delta}$
		34.40-34.94	34.47	ELE	$\alpha B_n+ nB_n$	
				E2MHE	$\alpha B_{2MH}+ 6B_{2MH}$	
				E*i*BuE	αB_{iBu}	
			34.22	EP*PE+EP*PE	$1,4\text{-}\alpha'B_1$(between E)	$S_{\delta\alpha\beta\delta}$
25	35.1-35.6					
		35.07	34.98	E*i*BuE	brB_{iBu}	
			34.99	PPP*	$1,2\text{-}brB_1$	$T_{\alpha\beta}$
		35.2-35.8	34.72	PP*PP	$1,4\text{-}\alpha'B_1$	$S_{\gamma\alpha\beta\delta}$
26	37.13-37.55					

	37.37	37.0-37.7	36.91	EPE EPEE	αB_1	$S_{\alpha\delta}$
27	37.71	37.77	37.41	PPEPmm<br>PEPPmm	$\alpha\gamma B_1$	$S_{\alpha\gamma}$
28	37.8-38.0					
		37.91	37.06	PP*P	1,2-brB$_1$	$T_{\alpha\gamma}$
		38.00	37.05	ELE+E2MHE	brBn+brB2MH	
29	38.05-38.25	38.23	37.16	PEPEr	$\alpha' B_1$	$S_{\alpha\gamma}$
				EEPPr	$\alpha' B_1$	
30	38.35	38.45	37.41	PEPP+PPEPmr+<br>mr	$\alpha' B_1$	$S_{\alpha\gamma}$
				PEPP+PPEPrr		
				PEPPmr+rm		
				PEPPrr		
31	39.27	39.35	39.35	E2MHE	$3B_{2MH}$	
32	45.2-47.6	45.0-47.5				
			41.17	P*PPP*		$S_{\delta\alpha\alpha\beta}$
			41.42	PPPP*		$S_{\gamma\alpha\alpha\beta}$
			43.86	EPPE		$S_{\alpha\alpha}$
			44.11	PPPE		
		46.74	44.36	PPPrr		
			43.86		$3B_{iBu}$	

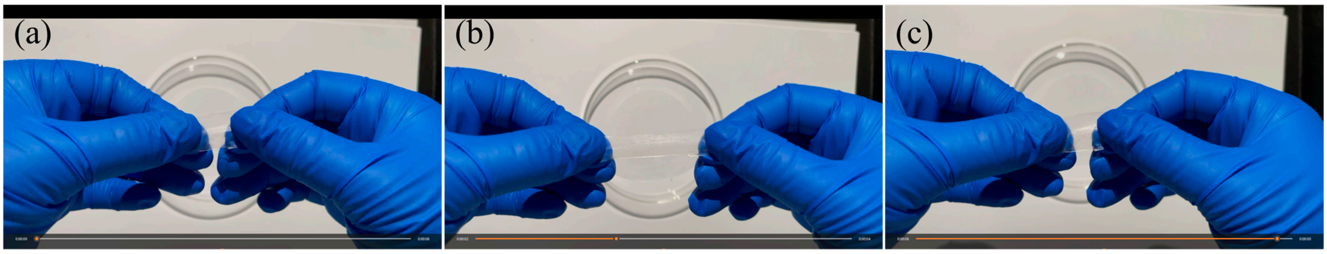

Figure S11 The stretching of polypropylene

Table S2 Mechanical properties of polypropylenes prepared by **C1** (Entries 4, 5, 6 in Table 1)

Sample	*T*<br>ºC	Tensile Strength<br>(MPa)	Young's Modulus<br>(MPa)	Elongation Break<br>(%)
1	0	1.03	2.19	218
2	30	0.75	1.46	317
3	50	0.29	1.20	403
